# A Narrative Review of Osteoid Osteomas and an Audit on the Practice of CT-Guided Radiofrequency Ablation in the Northern Irish Population: A First in the Literature

**DOI:** 10.7759/cureus.42034

**Published:** 2023-07-17

**Authors:** Sally Kamil, Eva Sweeney, Nagy Darwish

**Affiliations:** 1 School of Medicine, Dentistry and Biomedical Sciences, Queen's University Belfast, Belfast, GBR; 2 Care of the Elderly, Glan Clwyd Hospital, Rhyl, GBR; 3 Trauma and Orthopaedics, Belfast Health and Social Care Trust, Belfast, GBR

**Keywords:** ct-guided radiofrequency ablation, ct-guided percutaneous thermal ablation, osteoid osteoma, belfast, northern ireland, minimally invasive interventional radiology

## Abstract

Introduction

Osteoid osteoma is a benign condition of the bone, usually affecting young males. This retrospective study explores the demographics of osteoid osteomas in the Northern Irish population. It also aims to audit the practice of CT-guided radiofrequency ablation of osteoid osteomas at a major orthopaedic centre in Belfast, Northern Ireland, and to investigate the possible causes of treatment failure.

Methods

Forty-seven osteoid osteoma patients, diagnosed based on clinico-radiologic features and treated with CT-guided radiofrequency ablation, were found eligible for inclusion and analysis. We collected data from electronic health records (March 2011 to May 2022) and reviewed the radiological images and associated reports. Information about demographics, clinical indices, operative technique, clinical outcomes, biopsy results, and follow-up were also gathered. Data were then analysed using IBM SPSS Statistics for Mac, version 28.0.1.1 (14) (IBM Corp., Armonk, NY).

Results

The average age of patients was 19.3 years, with a male-to-female predilection of 2.1:1. The proximal and mid-tibial shafts were the most frequently involved sites. On average, patients had symptoms for 15.6 months, while the mean treatment delay period was 6.9 months. Primary clinical success was observed in 37 patients (78.7%), while ten patients had a clinical failure. Two out of the 10 patients with treatment failure underwent subsequent successful ablations, raising the secondary clinical success rate to (83.0%). Chi-Square association tests found no correlation between primary treatment outcomes and other qualitative variables (gender, bone type, lesion location, and Kayser classification). Moreover, binary logistic regression tests found no predictability of age and treatment delay on treatment outcomes. The overall observed complication rate was 4%, with only one significant side effect reported (third-degree skin burn).

Conclusion

We concluded that the demographics of osteoid osteomas in the Northern Irish population are comparable to what is previously established in the literature. Furthermore, we reasoned that CT-guided radiofrequency ablation is an efficient, safe, and effective minimally invasive technique in the management of osteoid osteomas.

## Introduction

Osteoid osteoma (OO) is a type of osteogenic benign bone tumour, originally defined as a clinical entity in 1935 by Henry Jaffe, a pathologist at The Hospital for Joint Diseases in New York, USA [1]. It accounts for around 2-3% of all primary bone neoplasms [2]. The rate of occurrence in the Northern Irish population has not been documented in the literature, and to our knowledge, only three publications on OOs in this population exist [3-5].

Epidemiology

Osteoid osteomas usually affect people aged 5 to 24 and have a male-to-female predilection of 3:1 [6]. Mario Campanacci, professor of Orthopaedic Surgery and Pathology at the University of Bologna in Italy, reviewed 836 OOs and mapped their distribution in the human skeleton. He found that OOs frequently occur as solitary lesions, are more prevalent in the appendicular skeleton, and seldom arise in the skull bones [7]. A preponderance of long bones, particularly of the lower limbs, has been reported in the literature, whereby approximately half of all these lesions develop in the femur and tibia, with the femoral neck being the most common site [2]. In the feet, they are more likely to occur in the talus [2]. As for the upper limb, they are most commonly observed in the humerus, followed by the ulna and radius [8]. The spine is affected in 11% of cases, with the posterior elements (neural arch) most commonly involved [6,9].

Classification

Osteoid osteomas can occur anywhere along the long bones; however, the diaphyses are more commonly involved. They typically occur in the cortex but may occur in other parts of the bone [10]. Kayser et al. (1998) classified OOs that occur in tubular bones into four types according to their location in such bones. In their study, Kayser et al. analysed computed tomography (CT) or magnetic resonance imaging (MRI) scans of 38 patients and categorised the lesions as follows: subperiosteal, intracortical, endosteal, and medullary [11].

Clinical presentation and natural history

The primary complaint is a dull nagging pain that worsens at night, which the patient may attribute to previous minor trauma. Initially, the pain is felt intermittently; however, as the disease progresses, the pain becomes more persistent, and the patient may start to localise a tender swelling. Sometimes the pain is not well-localised and may radiate or be referred to a nearby joint. Patients may report alcohol consumption as an aggravating factor, likely secondary to vasodilation. Very rarely, the lesion may be painless and detected incidentally on radiographs. In the spine, these lesions would lead to painful scoliosis secondary to muscle spasm reactive to the nidus and occasionally signs of nerve root impingement [12].

Gross pathology and histopathology

The osteoid osteoma is a benign small oval lesion with a diameter of less than 2 cm, differentiating it from an osteoblastoma. Grossly, the lesion is well-circumscribed with a centre (nidus) that encompasses a meshwork of osteoid and woven (primary or immature) bone trabeculae, with osteoblastic rimming, osteoclasts, and numerous blood vessels. The nidus is usually softer than the adjacent bone and is surrounded by varying degrees of reactive sclerosis [2,7].

Imaging

The typical clinical presentation aided by X-rays, in most patients, is sufficient to diagnose OOs, and at least two views centred over the concerned area should be obtained [13]. In specific skeletal locations, however, such as the axial skeleton and feet, overlapping bone shadows may obscure the lesion on plain radiographs, and more advanced imaging modalities are needed to establish the diagnosis [14]. Computed tomography (CT) is the best tool for the identification of the nidus, and ideally, it should be performed in thin slices (1-2 mm). It allows accurate localisation and classification of the lesion according to the Kayser et al. (1998) categorisation [15]. Typically, the OO nidus appears as a small (< 2 cm) rounded radiolucency, the centre of which may contain calcifications (punctate, amorphous, or ring-like), surrounded by variable degrees of radiodense reactive sclerosis producing the “bulls-eye” appearance. Magnetic resonance imaging (MRI) is poor in nidus identification compared to CT; nevertheless, in cases of unusual CT findings, MRI may provide additional information to aid the diagnosis [15].

Management

Medical therapy can be trialled in patients who opt for it, especially if they have a mildly painful OO, can tolerate prolonged medical treatment, or if the lesion is not amenable to intervention. Salicylates and non-steroidal anti-inflammatory drugs (NSAIDs) have long been employed in managing OOs with good outcomes, as around 75% of patients become pain-free by using incremental doses of aspirin [2]. During this time, OOs may regress spontaneously over two to six years [16]. Aside from that, surgical intervention to remove the nidus wholly is indicated.

Open surgical treatment can be offered to patients who opt for it or in cases of failure of conservative management. Classically, two surgical techniques, en bloc resection and curettage, have been described which aim to excise the OO nidus entirely to prevent local recurrence [16]. A success rate of 88% to 100% has been reported for en bloc resection [17]; however, several disadvantages of this technique, namely, prolonged hospitalisation, the possible need for postoperative rehabilitation, and the risk of insufficiency fractures at the surgical site have made it less appealing for patients and physicians [16].

Nowadays, surgical treatment is rarely used, and physicians opt for minimally invasive techniques (MIT) such as CT-guided percutaneous excision, CT-guided radiofrequency ablation (CT-RFA), CT-guided cryoablation, and CT-guided microwave ablation [16]. Percutaneous CT-guided radiofrequency ablation is one MIT, which some authors believe should be the first line of treatment [18]. Not only is this procedure of low risk and high success rate that reached 95% in one systematic review [19], but following CT-RFA, patients can resume day-to-day activities promptly without the need for a cast, splint, or any other mode of external support [18].

Although several MITs in the management of OOs have emerged, CT-RFA is currently considered the gold standard treatment. Nevertheless, treatment failures and complications have been reported in the literature, in addition to heterogeneity in the practice between different institutions [20].

This study aims to research the demographics of OOs in the Northern Irish population and to audit the practice of CT-RFA of OOs at a major orthopaedic centre in Belfast. We will also investigate the possible causes of the clinical failure of CT-RFA. This article was previously presented as a meeting abstract at the European Conference on Interventional Oncology 2023 in Stockholm, Sweden, held between April 16-19, 2023.

## Materials and methods

The audit department at Belfast Health and Social Care Trust/The Royal Victoria Hospital Trauma and Orthopaedics Research Group (TORG) provided permission for data collection and analysis. An IRB review and IRB approval was not required.

Patient population

**Figure 1:**
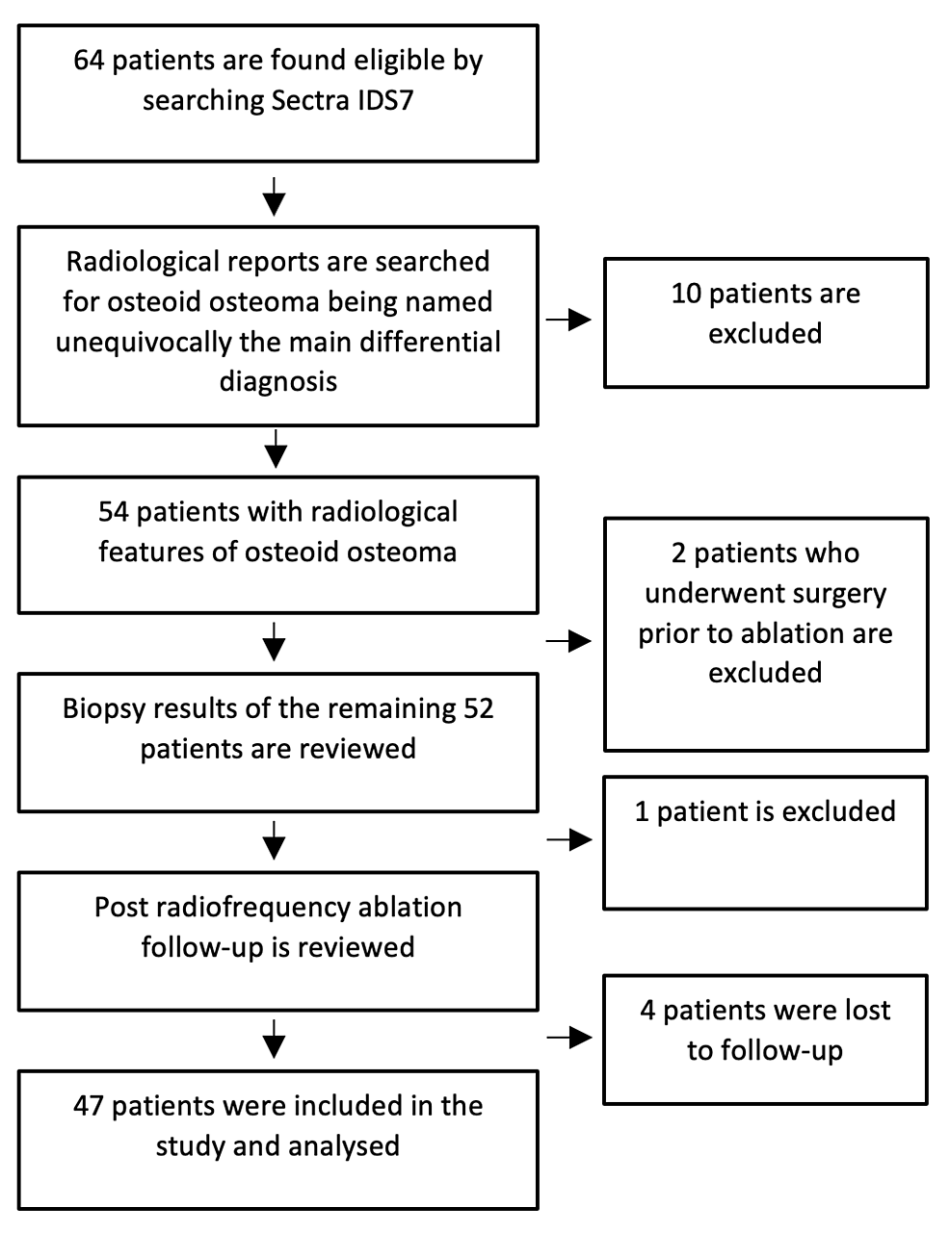
A flowchart illustrating the patient recruitment process

The picture archiving and communication system (PACS) Sectra IDS7 at Musgrave Park Hospital in Belfast, Northern Ireland, was searched retrospectively for CT-RFA patients over 10 years (March 2011 until May 2022). Sixty-four patients were found initially eligible, and the radiological images and associated reports were reviewed, looking for cases where “osteoid osteoma” was named the primary diagnosis. Therefore, seven patients were excluded as their principal differential diagnosis was otherwise (four chondroblastomas, one chondroid lesion, one chronic osteomyelitis, and one non-specific lesion). Furthermore, three patients’ diagnoses from imaging were borderline between an OO and an osteoblastoma, while two others had undergone surgical intervention before RFA; therefore, all five were also excluded. Following that, the biopsy results from the CT-RFAs were reviewed, and one patient who had a biopsy result confirming a diagnosis of osteoblastoma was excluded; however, a negative or inconclusive biopsy was not a criterion for exclusion. Finally, four patients were lost to follow-up, producing a final sample of 47. All the patients' imaging and electronic health records (EHR) were reviewed and analysed retrospectively (Figure 1).

Clinical and radiologic assessment

The following information was extrapolated from the PACS and EHRs: gender, age at diagnosis, signs and symptoms, duration of symptoms, lesion side and location, imaging modality used in diagnosis, the period from diagnosis to CT-RFA (treatment delay), ablation dose, admission time, technical outcomes, procedural complications, biopsy results, clinical outcomes, and follow-up details. In four patients, the exact date of symptom onset was unknown and thus presumed to be one month before their first imaging. In the long bones, the lesions were classified retrospectively according to the Kayser classification by a consultant musculoskeletal radiologist (AC) experienced in image interpretation, which was done on axial CT cuts (0.5-2 mm).

Operative technique

**Figure 2:**
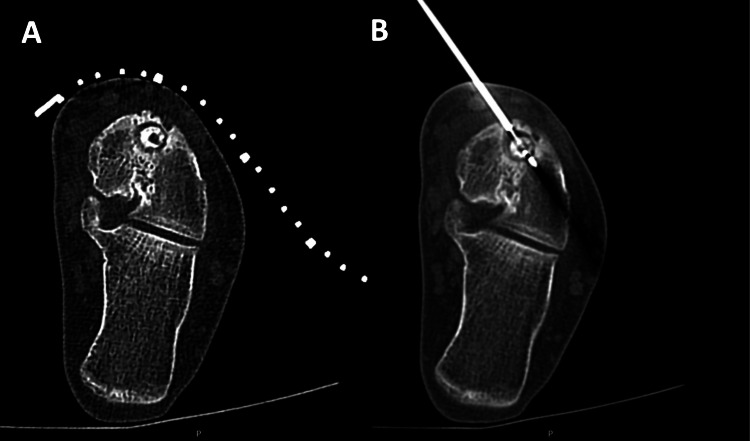
Scans showing CT-guided radiofrequency ablation of an osteoid osteoma in the neck of the talus bone of the left foot A, a pre-ablation scan showing the overlying adhesive localizer grid. B, a peri-ablation scan showing the radiofrequency ablation probe tip positioned inside the nidus. This is a computed tomography (CT) image of one of the patients included in the study.

All the CT-RFAs were performed by two consultant musculoskeletal radiologists (ME and NN) skilled at such interventions, and informed consent was obtained from the subjects. The ablations were performed in the procedural CT suite under general and local anaesthesia (1% lidocaine and/or 5% levobupivacaine) to guarantee patient immobilisation. The patient was positioned on the CT scanner bed, a small adhesive localiser grid was applied over the estimated lesion site, and a pre-procedural CT scan (slice thickness: 0.6-3 mm) was performed. For scans performed before 2015, a Siemens Somatom Sensation 16 (Germany) was used, while for those succeeding that, a Siemens Somatom Definition AS (Germany) was employed. The needle entrance site was marked on the skin choosing a path avoiding neurovascular structures and preferably following a perpendicular axis on the underlying bone, thus minimising the risk of the needle skidding off. After that, the grid was removed, the skin was prepped and draped under the aseptic technique, and grounding pads were applied to the patient’s skin. Next, under CT fluoroscopic guidance, a 14G penetration set (Bonopty Bone Biopsy System, Apriomed, Uppsala, Sweden) was used to enter the nidus, and a biopsy was attempted using a 15G biopsy set (Bonopty Bone Biopsy System, Apriomed, Uppsala, Sweden). The biopsy set was then removed, and a monopolar single RFA electrode was introduced into the nidus (RFA1507 or RFA1510, Covidien Cool-tip RF Ablation System E Series, Mansfield MA, USA), which was then connected to the monopolar RFA generator (200 Watts, Covidien Cool-tip RF Ablation System E Series, Mansfield MA, USA) on a temperature ablation setting. After the electrode was confirmed to be inside the nidus, an ablation temperature of 80-90 °C was targeted for 5-6 minutes (considered a technical success (Figures 2A, 2B); however, ablation temperatures and durations occasionally changed according to nidus size or in case of re-ablation. Once the radiologist was satisfied with the technical success of the procedure, the electrode and penetration set were removed, and a dressing was applied over the puncture site. Patients were then observed in the recovery room and were later discharged once appropriate. 

Patient follow-up

All the patients included in the author’s study presented for outpatient follow-up with the primary referring orthopaedic surgeon. Follow-up periods were heterogenous according to the surgeon’s experience; however, patients were only discharged from the service if the outcome was satisfactory. They were asked to return to the service if they had persisting or recurring symptoms. Post-ablation imaging, if available, was also reviewed.

Clinical success and clinical failure definitions

Clinical success was defined as the absence of symptoms one month following the procedure and without recurrence. On the other hand, clinical failure was defined as symptoms persisting more than two weeks following the procedure or recurring at any time following a pain-free interval. 

Data analysis

Data were analysed using IBM SPSS Statistics for Mac, version 28.0.1.1 (14) (IBM Corp., Armonk, NY). Chi-square association tests were performed to find whether a correlation between primary treatment outcomes and other qualitative variables (gender, bone type, lesion location, and Kayser classification) exists. Moreover, binary logistic regression tests were conducted between treatment outcomes, patient age, and treatment delay. An alpha (α) of 0.05 was considered statistically significant.

## Results

**Figure 3:**
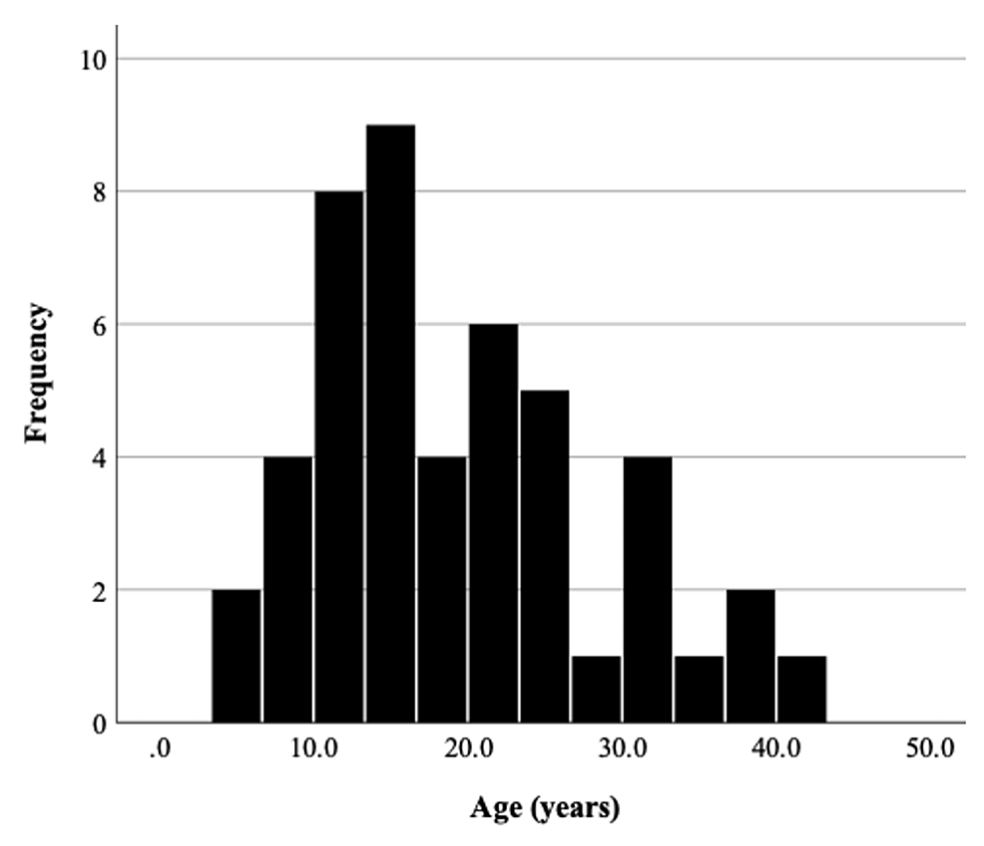
The distribution of osteoid osteomas in different age groups in our study

**Figure 4:**
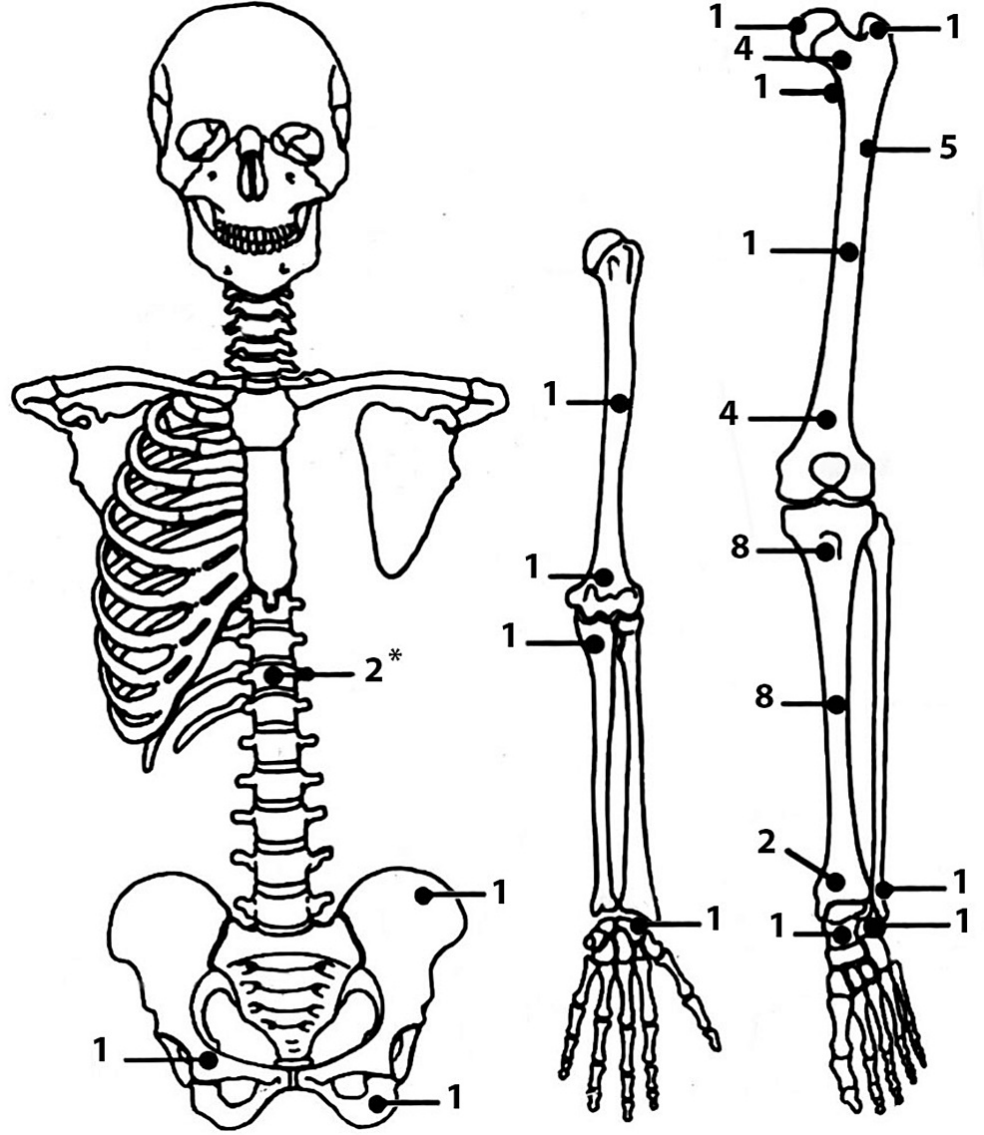
The skeletal distribution of osteoid osteomas in our study The numbers indicate the total number of lesions at different sites within our patient population. (*) thoracic spine lesions involve the superior and inferior articular facets. The sketch is drawn by one of the authors, SK.

We recruited 47 patients (32 males and 15 females), yielding a male-to-female predilection of 2.1:1. The average age of patients was 19.3 years, with ages ranging from 4.0 to 42.3 years (median age = 17.4 years) (Figure 3). Twenty-eight lesions were on the right side of the body, while 19 were on the left side. The distribution of the lesions is shown in Figure 4. In our study, the proximal and mid-tibial shafts were the most frequently involved sites. On average, patients had symptoms for 15.6 months (range = 1.1 to 82.4 months) before the diagnosis was established.

Out of 47 lesions, 39 were in long bones, and these were classified according to the Kayser classification: intracortical (56.4%), endosteal (23.1%), subperiosteal (15.4%), and medullary (5.1%). 

Treatment delay averaged 6.9 months (range = 0.4 to 58.7 months), upon which 47 patients underwent a primary ablation. A biopsy was attempted during all primary ablations; however, only 41 samples were successfully obtained, of which 22 were positive for OO (53.7%). Technical success was 100%, and only one complication was observed (third-degree skin burn) in a patient with a tibial mid-shaft OO. Primary clinical success was observed in 37 patients (78.7%), while ten patients had a clinical failure. A secondary ablation was performed in only two patients at 10.5 and 21 months following the primary ablation, raising the secondary clinical success rate to 83.0%. A biopsy was obtained in only one of the secondary ablations, which confirmed OO on histological examination. Like the primary ablations, technical success was observed in both cases. One of the procedures was complicated by a metallic fragment dislodging inside the bone, but this was not problematic for the patient. Tables 1, 2 illustrate the outcomes of the primary and secondary ablations, while Table 3 shows the biopsy results.

**Table 1:** Shows the outcomes of the primary ablations

Primary Ablations (n = 47)	Technical Success	Primary Clinical Success		Primary Clinical Failure
Number of Cases (%)	47 (100%)	37 (78.7%)		10 (21.3%)

**Table 2:** Shows the outcomes of the secondary ablations

Secondary Ablations (n = 2)	Technical Success	Secondary Clinical Success		Secondary Clinical Failure
Number of Cases (%)	2 (100%)	39 (83.0%)		8 (17.0%)

**Table 3:** Shows the biopsy results from the primary and secondary ablations

	Number of Biopsies Obtained	Positive Biopsy (%)	Negative or Inconclusive Biopsy (%)
Primary Ablations (n = 47)	41	22 (53.7%)	19 (46.3%)
Secondary Ablations (n = 2)	1	1 (100%)	0 (0%)
Total	42	23 (54.8%)	19 (45.2%)

Four of the 10 patients with primary clinical failure had persistent pain, while six had recurrent pain. Only four had histological confirmation of an OO, five had a negative biopsy, and in one case a sample was not sent. All 10 patients had postoperative imaging by CT and/or MRI to assess for residual or recurring OO on follow-up. Table 4 illustrates these patients' demographics, clinical indices, clinical outcomes, and follow-up details.

**Table 4:** Demographics, clinical parameters, and clinical outcomes of the 10 patients with primary clinical failure

Patient	Age (years), Gender	Lesion Location	Kayser Classification	Treatment Delay (months)	Primary Outcome	Biopsy Result	Residual Lesion on Imaging	Redo CT-RFA	Final Outcome
1	17.4, F	Proximal tibia, R	Endosteal	1.8	Persistent pain	Positive	No (MRI)	No	Self-referred to pain management service
2	13.5, M	Mid femur, R	Intracortical	3.6	Persistent pain	Positive	Yes (MRI)	Yes	Pain resolved immediately postoperatively
3	32.5, M	Proximal tibia, L	Intracortical	6.5	Persistent pain	Negative	No (stress fracture at ablation site on CT + MRI)	No (Activity limitation and low-intensity pulsed ultrasound)	Pain gradually resolved at 27.1 months follow-up
4	37.3, M	Distal tibia, L	Intracortical	5.0	Persistent pain	Negative	No (CT + MRI)	No	Pain gradually resolved at 27.5 months follow-up
5	8.1, F	Femur neck, R	Intracortical	2.0	Recurrent pain after 4.5 months	Negative	Yes (CT + MRI)	Yes	Pain resolved immediately postoperatively
6	13.7, F	Talus, L	N/A	15.1	Recurrent pain after 4.0 months	Positive	No (CT + MRI)	No	Pain gradually resolved at 36.0 months follow-up
7	24.0, M	Distal femur, L	Endosteal	4.6	Recurrent pain after 2.5 months	Positive	No (CT + MRI)	No	Pain stabilized at 19.1 months follow-up
8	37.4, F	Proximal tibia, R	Intracortical	11.0	Recurrent pain after 9.5 months	Negative	No (CT)	No	Pain still present at 13.4 months follow-up, but manageable by oral analgesics
9	8.0, M	Femur neck, R	Intracortical	1.3	Recurrent pain after 6.0 months	Negative	No (CT + MRI)	No	Pain gradually resolved at 23.5 months follow-up
10	18.4, M	T7 inferior articular process, R	N/A	4.8	Recurrent pain after 9.5 months	Not Sent	Yes (CT + MRI)	No	Pain still present at 19.0 months follow-up, and requires increasing doses of oral analgesics

Upon conduction of likelihood-ratio Chi-square tests (G^2^), no correlation was found between primary treatment outcomes, sex, bone type, lesion location, and Kayser classification (Table 5). In addition, logistic regression tests found no predictability of the patients’ ages or treatment delay on primary treatment outcomes. 

**Table 5:** The results of the likelihood ratio Chi-square tests (G2) correlating primary treatment outcomes with other variables

Correlation	G^2^	Degrees of Freedom (df)	p-value
Gender & Outcome	0.372	1	0.542
Bone Type & Outcome	0.077	1	0.781
Lesion Location & Outcome	0.845	1	0.358
Kayser Classification & Outcome	4.340	4	0.362

As for admission times, 40 cases were performed as day-case procedures, six required an overnight stay, and only three needed a two-day stay in the hospital. Follow-up times and methods (e.g., performing imaging on follow-up) were heterogeneous depending on the referring surgeon’s experience. The average follow-up was 10.1 months, with a minimum period of one month and a maximum period of 36.0 months.

## Discussion

Study demographics 

Our study shows that OOs are more common in males than females, which is a fact already reflected in the literature. Although a reason for this predilection has not been previously proven, we believe it is due to a higher bone mass in males [21]. Most cases clustered between the ages of six and 26 years, an observation previously described in studies, which is likely secondary to greater bone growth during this period [22]. Like preceding research, the lower limbs were more commonly involved than the upper extremities, with 17 cases occurring in the femur and 18 in the tibia, collectively accounting for approximately 74% of cases. 

Establishing the diagnosis

In our study, we relied on the clinical and radiologic features in establishing the diagnosis. All patients who had imaging exhibited typical signs and symptoms of an OO. The argument for a per-procedural biopsy is controversial in the literature. Some authors relied on symptomology and imaging features in diagnosing OOs [23-26], while others considered histological confirmation essential for diagnosis [27,28]. This is because biopsy results are inconsistent in the literature (Table 6), likely secondary to insufficient sampling during the procedure [29,30]. Furthermore, histological confirmation is non-essential in the diagnosis of OOs due to the high specificity of imaging in validating the diagnosis [31]. In one systematic review and analysis of 36 studies on percutaneous thermal ablation of OOs, which included 1863 ablations in 1798 patients, a sample was obtained from all lesions in 13 analyses, while a biopsy was not performed on all or some lesions in 23 studies. The number of biopsies performed was 766, which yielded a positive result in 59.3% of cases, which is not a far figure from our result (54.8%). The remaining biopsies yielded normal bone tissue, non-diagnostic results, or were merely suggestive but not pathognomonic of an OO [31]. In our opinion, histological confirmation is unnecessary if the lesion is unequivocally an OO based on symptomatology and radiological features. However, a biopsy may be helpful in CT-RFA clinical failure [32].

**Table 6:** Compares biopsy findings of different interventional studies on osteoid osteomas CT-RFA, CT-guided radiofrequency ablation [7,12,29,30].

Study	Number of Patients	Histologic Confirmation	Intervention
Campanacci M (1999) [7]	97	100%	Surgical Excision
Unni and Inwards (2010) [12]	30	50%	CT-RFA
Somma et al. (2021) [29]	102	100%	CT-RFA
Baal et al. (2019) [30]	71	75%	CT-RFA

Treatment and outcomes 

At our orthopaedic centre, the period from diagnosis to CT-RFA averaged 6.9 months (range = 0.4 to 58.7 months), which showed no predictability on primary treatment outcomes upon conducting logistic regression tests. Although this is a positive finding, OO patients should be offered a definitive treatment as soon as possible to alleviate their symptoms. 

All procedures were technically successful, but primary clinical success was achieved in 37 out of 47 patients (78.7%). Patients’ ages and lesion locations (axial vs appendicular skeleton) did not influence initial treatment outcomes. This is similar to the findings of Tordjman et al. (2020) in their systematic review of 69 studies on CT-RFA of OOs [20]. Furthermore, the Kayser classification of the lesion had no association with primary clinical success or failure, which is a new finding not previously explored in the literature. Although no correlation between gender and the initial management outcomes was found, Baal et al. (2019) showed female gender as a risk factor for symptomatic recurrences following CT-RFA of OOs [30]. Only three true symptomatic recurrences (detected on imaging) existed in our population (two males and one female), precluding the possibility of making any credible conclusions. 

Compared to the published literature** **(Table 7)** **[23,24,26,27,29,33-35], our monopolar Cool-tip approach achieved slightly lower rates of primary clinical success. Although our secondary clinical success is 83%, we can argue that it is, in fact, higher than that figure, as repeat ablation was attempted in only two patients while it was not tried in the remaining eight patients. All the studies had attempted to repeat ablation on cases with a primary clinical failure, thus achieving a higher secondary clinical success than our report. Moreover, three of these studies had a tiny population size which precludes them from being valid studies for comparison. Perhaps the best reference for contrast is Tordjman et al.'s (2020) systematic review, which showed a primary clinical failure of 8.3%, while the secondary clinical failure was 3.1% [20]. 

**Table 7:** Previous CT-guided radiofrequency ablation studies of osteoid osteomas and their clinical outcomes. CT, Computed Tomography [23,24,26,27,29,33-35].

Study, Sample size (n)	Probe Type	Primary Clinical Success	Secondary Clinical Success
Rehnitz et al.(2013) [23], n = 72	Monopolar, Cool-tip	99%	100%
Cantwell et al. (2006) [24], n = 11	Monopolar, Cool-tip	100%	----
Chahal et al. (2017) [26], n = 87	Bipolar, Non-cool-tip	86.2%	96.5%
Morassi et al. (2014) [27], n = 13	Monopolar, Non-cool-tip	84.6%	100%
Somma et al.(2021) [29], n = 102	Bipolar, Cool-tip	96%	100%
Martel et al. (2009) [33], n= 10	Monopolar, Non-cool-tip	80%	100%
Lassalle et al. (2017) [34], n = 88	Monopolar, Cool-tip	89.8%	94.3%
Yuce et al. (2020) [35], n = 55	Monopolar, Cool-tip	96.4%	98%

One issue we must draw attention to is the heterogeneity of technical and clinical definitions between different studies, including ours, which may introduce errors when outcomes are compared. For example, in their analysis, Somma et al. (2021) considered a procedure technically successful if the probe was positioned inside the nidus so that no portion was more than 5 to 7 mm away from the uninsulated tip of the RFA electrode and if the target ablation dose was delivered. On the other hand, Niazi et al. (2021) defined technical success as positioning the probe tip within the nidus and depositing the target energy [36]. Yuce et al. (2020) also reported the outcomes of CT-RFA of OOs; however, they gave a loose definition for clinical success as ‘resolution of symptoms within one month after initial therapy’ and did not allude to the meaning of therapeutic failure [35]. Therefore, we recommend standardising definitions to allow more accurate comparisons between various analyses.

The ablation dose and equipment also influence treatment outcomes. In all patients with primary clinical failure, an ablation dose of 90 °C for at least 5-6 minutes was deployed, which falls within the recommended amount in the literature [32]. Therefore, we do not believe that this factor influenced the results. Moreover, we utilised Cool-tip RFA probes, which infuse saline through the electrode, thus generating larger currents with greater heat transmission and larger treatment zones. However, the ablation perimeter may be unpredictable at times and damage extra-nidal tissues. Although Cool-tip electrodes produce larger treatment zones, it is unclear from the published literature whether they yield superior clinical outcomes compared to non-cooled-tip probes. 

Upon examining the 10 patients with primary treatment failure, we found that only four had a biopsy-proven OO. Of these four patients, one had a subsequent ablation, one patient referred herself to a private pain management practice, and in the two remaining patients, the pain eventually resolved at 19.1 and 36.0 months follow-up. As OOs may resolve spontaneously after two to six years, the likely explanation in the two latter patients is the involution of the lesions following a prolonged observational period. In the remaining six patients with a negative biopsy, one patient had another ablation which resolved his symptoms, and one patient was found to have a stress fracture at the ablation site, which provides a rationalisation for his persistent pain. Two patients with negative biopsies saw their pain resolve after prolonged watchful waiting (27.1 and 27.5 months), representing probable regression of the lesions on their own. On the other hand, the last two patients with negative biopsies had ongoing pain at 13.4 and 19.0 months, which raises the question of whether the lesions were, in fact, an OO and whether the pain would have resolved had they been followed up for more extended periods.

As for complications, we observed a rate of 4% (one skin burn and one broken needle). Overall, our practice can be considered safe as the single skin burn was a significant side effect, while the broken needle caused no harm to the patient. In Tordjman et al.'s (2020) systematic review, a global complication rate of 3% was reported, with skin burns being the most frequent (0.7%) and broken needles less commonly observed (0.3%) [20]. The third-degree skin burns that we reported occurred following the ablation of a subperiosteal lesion located in the middle part of the anterior tibial shaft, which is subcutaneous and prone to skin burns during RFA as reported in the literature [32,37]. Likewise, the bones of the hand and feet are rather subcutaneous and liable for skin burns during thermal ablation. Therefore, special measures have been recommended in order to minimise such risk: maintaining a distance of 10 mm from the RFA electrode’s tip to the nearby skin, withdrawing the bone-penetration cannula 10 mm above the non-insulated tip of the ablation electrode, using RFA probes with smaller treatment zones, injection of 2% dextrose subcutaneously over the treatment zone, and application of sterile, cold saline-soaked sponges over the skin during ablation. In our series, no special measures had been applied during the ablation of the aforementioned lesion; however, a distance of more than 1 cm was maintained from the overlying skin. Radiological images to determine if the penetration cannula had been withdrawn during the ablation were not available. 

Similar to other studies, the ablations were essentially a day-case procedure, with the majority of patients discharged on the same day [32,37]. This reflects positively on cost and resources, pitching CT-RFA as an efficient treatment option.

Follow-up times in our analysis were heterogeneous and dependent on clinician experience, with an average follow-up of 10.1 months (range = one month to 36.0 months). Although some patients were discharged from the service as early as one month, they were instructed to return if they experienced a recurrence of symptoms. We believe that a timely follow-up schedule should be applied in practice. Following CT-RFA, patients usually become pain-free by the end of the first week [24,27], and a follow-up at two to four weeks is recommended [20,34], whereby patients who require a further ablation are recognised early. Unfortunately, the literature has not documented recommendations for follow-up intervals, and further research in this field should be encouraged. 

Limitations of the study

There are a few shortcomings to our study that we must acknowledge. First, the study’s retrospective nature inherently introduces recall and selection biases. Second, our inclusion criteria for the diagnosis of OO are still debated in the literature (clinico-radiological vs biopsy). Third, a scale, such as the visual analogue scale (VAS) for pain, was not used to quantify treatment response, but rather a vague terminology was used (pain resolved, persistent pain, recurrent pain, and pain stabilised). Finally, follow-up periods were variable, which may have influenced our results.

## Conclusions

Osteoid osteomas are rare benign bone lesions that more commonly affect young males and usually occur in the cortex of the long bones of the hindlimbs. They are diagnosed by observing typical radiological features in a patient presenting with characteristic signs and symptoms, and a biopsy is not essential for diagnosis. Computed tomography-guided radiofrequency ablation is a safe, efficient, and effective technique that should be offered as a first-line treatment for eligible patients. The patient’s age, gender, lesion location, and treatment delay period do not seem to influence treatment outcomes.
